# The independent association of mean platelet volume with overall survival in multiple myeloma

**DOI:** 10.18632/oncotarget.11551

**Published:** 2016-08-23

**Authors:** Qiang Zhuang, Lina Xiang, Hanyan Xu, Fang Fang, Chongyun Xing, Bin Liang, Kang Yu, Jianhua Feng

**Affiliations:** ^1^ Department of Hematology, The First Affiliated Hospital of Wenzhou Medical University, Wenzhou 325000, PR China; ^2^ Department of Emergency Medicine, The First Affiliated Hospital of Wenzhou Medical University, Wenzhou 325000, PR China; ^3^ Department of Pneumology, The First Affiliated Hospital of Wenzhou Medical University, Wenzhou 325000, PR China; ^4^ Department of Pediatric Hematology-Oncology, The First Affiliated Hospital of Wenzhou Medical University, Wenzhou 325000, PR China

**Keywords:** multiple myeloma, mean platelet volume, prognosis, survival

## Abstract

We retrospectively analyzed the association between mean platelet volume (MPV) and prognosis in 62 newly diagnosed multiple myeloma (MM) patients. The associations between MPV and clinical characteristics were assessed. The log-rank test and the Cox proportional hazards model were used to evaluate the effect of MPV on survival. A MPV value of 8.50 fl was considered to be the optimal cut-off value for prognosis. MPV was associated with IgA isotype (P=0.012), serum creatinine concentration > 176.8 μmol/L (P=0.025) and IgH rearrangement (P=0.008). The log-rank test demonstrated that patients with low MPV experienced a shorter overall survival (OS) (P=0.0397). The multivariate analysis demonstrated that low MPV was an independent prognostic factor for OS [hazard ratio (HR)=2.44, P=0.026]. Therefore, we demonstrated that low MPV predicted an unfavorable prognosis in patients with MM.

## INTRODUCTION

Multiple myeloma (MM) is a malignant disorder of plasma cells, associated with an increased thromboembolic risk, particularly venous thromboembolism (VTE) [1, 2]. Several underlying prothrombotic mechanisms in MM have been put forward. Except for patient related factors (such as advanced age, immobility and obesity), fibrin polymerization and fibrinolysis impairment, enhanced platelet adhesion and potential dysfunction, acquired resistance to activated protein C, as well as increased levels of inflammatory cytokines are considered as triggering factors for hypercoagulability [3]. It is also well established that anti-myeloma therapeutic regimens, such as chemotherapy and immunomodulatory agents (thalidomide and its derivatives lenalidomide and pomalidomide), increase the risk of thrombosis in patients with MM [3, 4]. Moreover, single nucleotide polymorphisms (SNPs) related to perturbed endothelium may also be a risk factor for the development of thrombosis in these patients [5]. On the other hand, in a large population-based study including over 9,000 MM patients, the occurrence of thrombosis was found to be associated with a significantly poorer survival [6]. Therefore, the incorporation of variables associated with risk of thrombosis into prognostic classification systems for MM may be available.

Mean platelet volume (MPV), which reflects platelet size, is a routinely determined parameter by complete blood count (CBC) analyzers [7]. Platelet size was proposed to be associated with platelet activity, and activated platelets play a significant role during thrombus formation and development. In fact, increased level of MPV has been found to be associated with various thromboembolic disorders in patients without cancer [8–10], while a decreased risk of VTE in patients with cancer [11]. Furthermore, VTE was noted to be correlated with poor survival in patients with cancer [12]. To the best of our knowledge, only one study has explored the prognostic significance of MPV in patients with MM: the study by Riedl et al. [11], who evaluated the data of 1,544 patients with various types of cancer (including 39 patients with MM) and found that high MPV values were associated with an improved patient survival. However, due to the relatively small sample sizes in subgroups of different types of cancer, no association of MPV with the risk of mortality could be found in individual cancer types, except for pancreatic cancer. Thus, the primary aim of this study was to investigate the prognostic effect of MPV prior to the initiation of treatment in a relatively large series of 62 patients with MM.

## RESULTS

### Patient population

The median age of the patients was 60.5 (37-78) years and the male to female ratio was 1.48:1. The most prevalent MM type was IgG (53.2%), and 24.2% of patients had light chain disease. Regarding ISS stage, 64.5% of patients had stage III, and 35.5% had stage I or II disease. In addition, the multiple probe fluorescent in situ hybridization (FISH) kit revealed that 46 (74.2%) patients had at least one abnormality. The most frequent abnormality was IgH rearrangement, which was seen in 40 (64.5%) patients. 1q21 amplification was observed in 35 (56.5%) cases, whereas both 13q14 deletion (D13S319 and/or RB1 deletion) and p53 deletion were detected in 12 (19.4%) cases. Other clinical and laboratory characteristics are summarized in Table 1.

**Table 1:** Baseline patient characteristics

Characteristics	Total (n=62)	Low MPV (≤ 8.50 fl) (n=22)	High MPV (> 8.50 fl) (n=40)	P-value
Age > 60 years	31/62 (50.0%)	13/22 (59.1%)	18/40 (45.0%)	0.288
Sex, male	37/62 (59.7%)	14/22 (63.6%)	23/40 (57.5%)	0.637
ECOG PS > 2	4/62 (6.5%)	2/22 (9.1%)	2/40 (5.0%)	0.610
Comorbidity				
Diabetes	12/62 (19.4%)	2/22 (9.1%)	10/40 (25.0%)	0.129
History of VTE	1/62 (1.6%)	0/22 (0)	1/40 (2.5%)	1.000
History of arterial event	3/62 (4.8%)	1/22 (4.5%)	2/40 (5.0%)	1.000
Complication				
Arterial thrombosis during follow-up	8/62 (12.9%)	5/22 (22.7%)	3/40 (7.5%)	0.119
ISS stage				0.914
I/II	22/62 (35.5%)	8/22 (36.4%)	14/40 (35.0%)	
III	40/62 (64.5%)	14/22 (63.6%)	26/40 (65.0%)	
Isotype				
IgG, κ, or λ	33/62 (53.2%)	9/22 (40.9%)	24/40 (60.0%)	0.149
IgA, κ, or λ	8/62 (12.9%)	6/22 (27.3%)	2/40 (5.0%)	0.012
Light chain disease	15/62 (24.2%)	3/22 (13.6%)	12/40 (30.0%)	0.150
others	6/62 (9.7%)	4/22 (18.2%)	2/40 (5.0%)	0.093
Hemoglobin < 100 g/L	41/62 (66.1%)	17/22 (77.3%)	24/40 (60.0%)	0.169
Platelets < 150×10^9^/L	31/62 (50.0%)	13/22 (59.1%)	18/40 (45.0%)	0.288
Creatinine > 176.8 μmol/L	8/62 (12.9%)	0/22 (0)	8/40 (20.0%)	0.025
Calcium > 2.75 mmol/L	5/51 (9.8%)	0/18 (0)	5/33 (15.2%)	0.082
Albumin < 35 g/L	34/62 (54.8%)	11/22 (50.0%)	23/40 (57.5%)	0.570
β2-microglobulin > 5.5 mg/L	29/62 (46.8%)	9/22 (40.9%)	20/40 (50.0%)	0.492
LDH ≥ (2× ULN)	1/57 (1.8%)	1/21 (4.8%)	0/36 (0)	0.368
BM plasma cell ≥ 30%	22/59 (37.3%)	7/22 (31.8%)	15/37 (40.5%)	0.503
Cytogenetics (FISH)				
1q21 amplification	35/62 (56.5%)	15/22 (68.2%)	20/40 (50.0%)	0.167
13q14 deletion	12/62 (19.4%)	5/22 (22.7%)	7/40 (17.5%)	0.618
p53 deletion	12/62 (19.4%)	4/22 (18.2%)	8/40 (20.0%)	0.862
IgH rearrangement	40/62 (6.5%)	19/22 (86.4%)	21/40 (52.5%)	0.008
Front-line treatment regimen				0.884
Novel drug-based	53/62 (85.5%)	19/22 (86.4%)	34/40 (85.0%)	
Older drug-based	9/62 (14.5%)	3/22 (13.6%)	6/40 (15.0%)	
SCT	8/62 (12.9%)	3/22 (13.6%)	5/40 (12.5%)	0.898

MPV, mean platelet volume; ECOG, Eastern Cooperative Oncology Group; PS, performance status; VTE, venous thromboembolism; ISS, international staging system; LDH, lactate dehydrogenase; ULN, upper limit of normal value; FISH, fluorescent in situ hybridization; BM, bone marrow; SCT, stem cell transplantation.

The median follow up for the entire cohort of 62 patients was 42 (5-86) months. A total of 8 (12.9%) patients developed arterial thrombosis during follow-up. A total of 27 deaths had been recorded by the time of the last follow-up. Median value for OS was 56.7 months.

### Optimal cut-off values for MPV and correlation between MPV and clinical characteristics

The ROC curve for MPV was used to determine the cut-off values. The area under the curve for MPV was 0.656 [95% confidence interval (CI): 0.515-0.797]. An MPV of 8.50 fl corresponded to the maximum joint sensitivity and specificity on the ROC curve (55.56% sensitivity and 80.0% specificity). The associations of MPV with clinical characteristics in this study population are shown in Table 1. The results showed that only IgA M component, creatinine and IgH rearrangement were associated with MPV level. The percentage of patients with IgA M component in low MPV (≤ 8.50 fl) cases was 27.3%, significantly higher than that (5.0%) in high MPV (> 8.50 fl) cases (P=0.012). The percentage of patients with serum creatinine concentration > 176.8 μmol/L in low MPV cases was 0, significantly lower than that (20.0%) in high MPV cases (P=0.025). In addition, the frequency of IgH rearrangement in low MPV cases was 86.4%, significantly higher than that (52.5%) in high MPV cases (P=0.008). Although low MPV was more correlated with more arterial thrombotic events developed during follow-up, no statistical significance was observed (P=0.119).

### Prognostic impact of MPV in patients with MM

**Figure 1:**
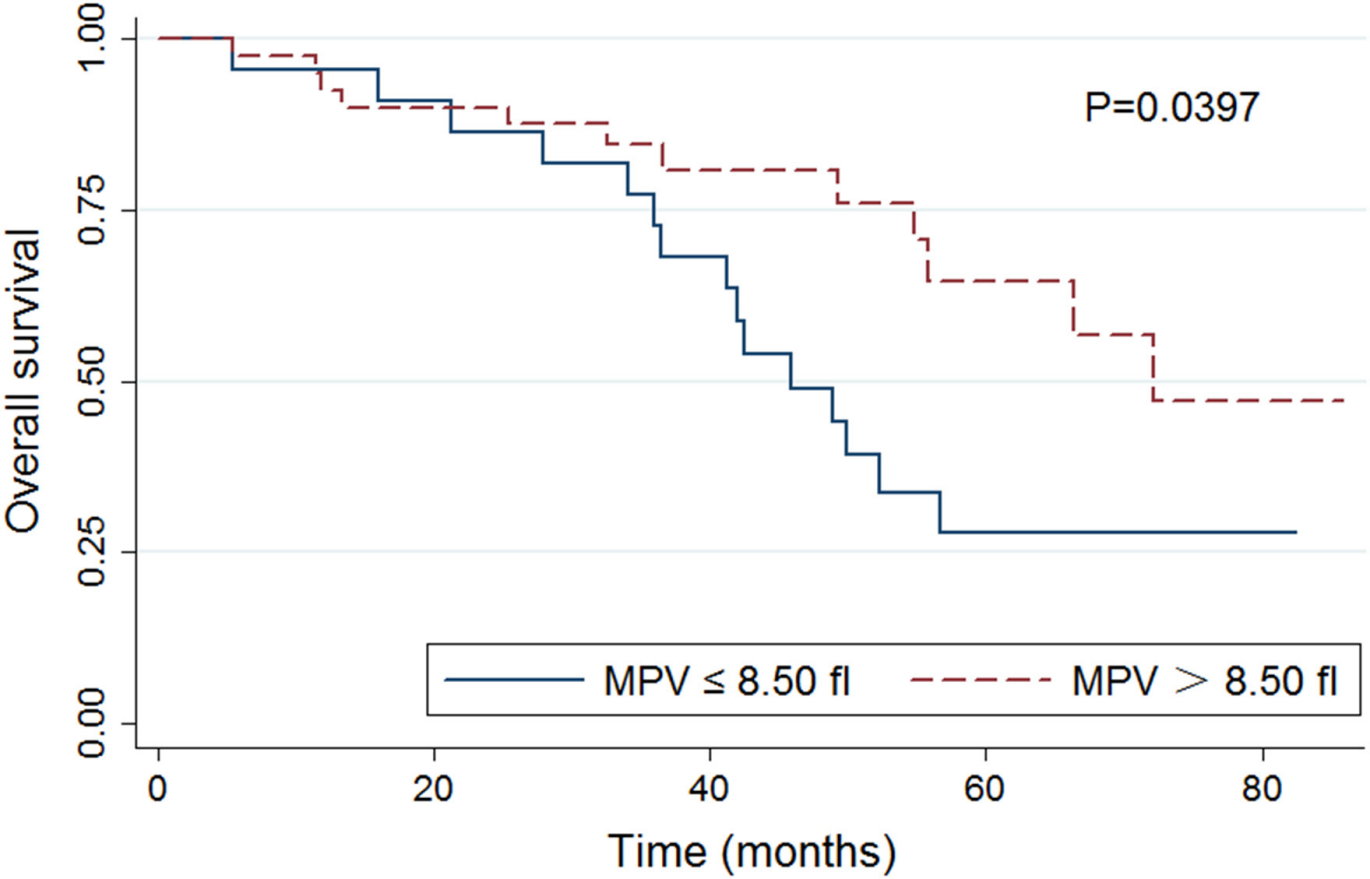
Kaplan-Meier analysis of overall survival of all the patients included in this study stratified by mean platelet volume (MPV; ≤8.50 vs. >8.50 fl)

The Kaplan-Meier analysis was performed to determine whether MPV was associated with overall survival (OS). The OS was significantly shorter in the group with a MPV of ≤ 8.50 fl compared to that in the group with a MPV of > 8.50 fl (P=0.0397; Figure 1). Results of the univariate and multivariate analysis for factors influencing OS in patients with MM are reported in Table 2. The univariate analysis showed the following clinical parameters were significantly associated with OS: MPV and the percentage of BM plasma cell. While the relationship between older age (> 60 years) and OS approached, but did not reach, statistical significance (P=0.050). Multivariate analysis that included all the parameters having a P value of less than 0.1 in the univariate analysis revealed that the low MPV was independently associated with shorter OS (HR=2.44, 95% CI 1.11-5.38, P=0.026). The higher proportion of BM plasma cell (>30%) was also shown to be independent prognostic factors for the OS.

**Table 2:** Univariate and multivariable analyses for overall survival

Variables	Univariate analysis	Multivariate analysis	
	P-value	HR (95% CI)	P-value
Low MPV	0.045	2.44 (1.11-5.38)	0.026
Age > 60 years	0.050	1.52 (0.68-3.38)	0.311
Male	0.851		
ECOG PS > 2	0.180		
Comorbidity			
Diabetes	0.746		
History of thrombosis^a^	0.434		
Complication			
Arterial thrombosis during follow-up	0.328		
ISS stage III	0.584		
Hemoglobin < 100 g/L	0.814		
Platelets < 150×10^9^/L	0.486		
Creatinine > 176.8 umol/L	0.614		
Calcium > 2.75 mmol/L	0.470		
Albumin < 35 g/L	0.896		
β2-microglobulin > 5.5 ug/mL	0.509		
LDH ≥ (2× ULN)	1.000		
BM plasma cell ≥ 30%	0.003	3.69 (1.58-8.60)	0.002
Cytogenetics (FISH)			
1q21 amplification	0.283		
13q14 deletion	0.962		
p53 deletion	0.327		
IgH rearrangement	0.205		
Novel drug-based treatment regimen	0.664		
SCT	0.766		

MPV, mean platelet volume; ECOG, Eastern Cooperative Oncology Group; PS, performance status; ISS, international staging system; LDH, lactate dehydrogenase; ULN, upper limit of normal value; BM, bone marrow; FISH, fluorescent in situ hybridization; SCT, stem cell transplantation; HR, hazard ratio; 95% CI, 95% confidence interval.

a including the history of VTE and arterial event.

## DISCUSSION

Platelets have been suggested to play an important role in cancer progression and metastasis [13]. A previous study demonstrated that preliminarily activated platelets have tumor-promoting properties [14]. More relatively, recent clinical reports demonstrated the negative effect of low MPV on the prognosis of cancer patients [11]. The present study including 62 patients with MM demonstrated that low MPV prior to the initiation of treatment was associated with decreased OS in patients with MM, which supports the findings of previous studies. To the best of our knowledge, this is the first study to demonstrate the prognostic effect of MPV on OS in patients with MM.

MPV, as an indicator of platelet activity [15], has been shown to be associated with platelet aggregation [16], thromboxane B2 production [17] and increased surface expression of the membrane glycoprotein IIb/IIIa complex [18]. Considering that the tendency of larger platelets to react to stimuli [19], thus it may be key to explaining the observations that increased MPV is associated with thromboembolic diseases in patients without cancer, such as myocardial infarction, stroke, or unprovoked VTE [8–10]. However, contrary to the observations in patients without cancer, in one lager, prospective, observational cohort study of cancer patients, high MPV was found to be associated with a decreased risk of VTE in patients with cancer [11]. One theory proposed to explain this phenomenon is that smaller platelets might exhibit a stronger pro-thrombotic tendency than larger platelets in cancer patients. This hypothesis was supported by the date from one retrospective study analyzing MPV levels in cancer patients who developed VTE [20]. It was found that MPV values were lower at the time of thrombosis development compared to those measured at the time of cancer diagnosis. Considering that patients with MM have an increased risk of thromboembolic events [1, 2], and what's more, the development of thrombosis was reported to be associated with poor survival in a large population-based study of MM patients [6], not surprisingly then, low MPV might reflect high thromboembolic risk, which in turn correlates with poor clinical outcome in patients with MM.

One of the limitations of the present study was the retrospective nature, thus caution should be taken when interpreting the results of this study because of the selection bias. For example, in contrast to the previously published population-based study based on over 9000 MM patients [6], our results showed that the development of arterial thrombosis during follow-up was not associated with survival in MM (Table 2). Apart from the potential inaccuracy of thrombotic diagnosis obtained from the review of clinical records, the heterogeneity in terms of study design, patient selection and treatment might also account for the diversity. Moreover, only certain types of IgH translocation including t(4;14) and t(14;16) have been identified to be associated with prognosis in MM [21–23]. Unfortunately, in this retrospective study, the translocation of IgH was detected by an IgH break-apart rearrangement probe but not by t(4;14) and t(14;16) probes due to the high fee. Furthermore, we had no complete and detailed information on the influence factors for MPV, such as the smoking behavior for the total cohort [24, 25], thus we could not adjust the association of MPV with risk of death for these influence factors.

In summary, this study demonstrated that low MPV prior to the initiation of treatment was an independent unfavorable prognostic factor in MM patients. MPV, available in a routine CBC examination, may represent one of the easiest measurements to be used as a prognostic marker in patients with MM. Further investigations are required to elucidate the precise mechanisms underlying the influence of platelet size on thrombosis development in patients with MM, as well as the prognostic effect of MPV in MM.

## MATERIALS AND METHODS

### Study population

This retrospective study analyzed the records of 62 patients with newly diagnosed MM, and all patients were treated at the First Affiliated Hospital of Wenzhou Medical University between February 2009 and December 2013. Patients who had the full clinical information including laboratory parameters (immunoglobulin type of monoclonal protein, hemoglobin, platelet count, creatinine, calcium, albumin, β2-microglobulin, lactate dehydrogenase, bone marrow plasma cells, and MPV) before any therapy were included. Besides, information on comorbidities (such as diabetes, and history of VTE and arterial event [coronary artery disease and cerebrovascular disease]) as well as the complications (such as the development of thrombosis during follow-up) was obtained. For cytogenetics analysis, FISH was performed in all patients using the following probes: 1q21 probe, D13S319 (13q14.3) probe, RB1 (13q14) probe, p53 (17p13.1) probe, and IgH (14q32) break-apart rearrangement probe. All probes were purchased from Peking Ginpujia Medical Technologies. Patients who were diagnosed with monoclonal gammopathy of undetermined significance, asymptomatic MM, amyloidosis, and plasma cell leukemia were excluded. Patients with sepsis at diagnosis were also excluded from the study. Written informed consent was obtained from each patient before entering the study according to the Declaration of Helsinki, and the study was approved by the Institutional ethics committee.

According to the patients' economic situation, novel drug-based (mainly bortezomib and thalidomide) or older drug-based (mainly anthracylines) regimens were used. In total, 37 patients (59.7%) received a bortezomib-containing regimen as first-line therapy: PAD (bortezomib, adriamycin and dexamethasone), VD (bortezomib and dexamethasone) and PCD (bortezomib, cyclophosphamide and dexamethasone); 5 patients (8.1%) received a thalidomide-containing regimen as first-line chemotherapy: TD (thalidomide and dexamethasone), TCD (thalidomide, cyclophosphamide and dexamethasone) and MPT (melphalan, prednisolone and thalidomide); 11 patients (17.7%) were treated with VTD (bortezomib, thalidomide and dexamethasone); 9 patients (14.5%) received older drug regimens as first-line chemotherapy: VAD (vincristine, adriamycin and dexamethasone). In addition, 6 patients (9.7%) underwent autologous stem cell transplantation and 2 (3.2%) received allogeneic stem cell transplantation following at least 4 cycles of treatment.

### Statistical analysis

The chi-square test or Fisher's exact test for discrete variables was used to compare patient characteristics. Receiver operating characteristics (ROC) curve were derived from the MPV value and survival status. In a ROC curve, the sensitivity and specificity were calculated by combining the optimal cut-off value for MPV and survival outcome. OS was defined as the period from the date of diagnosis to the date of the last follow-up or death from any cause. OS was evaluated through the Kaplan-Meier estimates and compared through the log-rank test. The Cox proportional hazards model was used for univariate analysis. Covariates having a P value of less than 0.1 in the univariate analysis were included in the multivariate Cox proportional hazards model. All statistical analyses were performed using Stata version 12 software (College Station, TX). A P value <0.05 was considered significant for all analyses.
